# Sub-ppm Methane Detection with Mid-Infrared Slot Waveguides

**DOI:** 10.1021/acsphotonics.3c01085

**Published:** 2023-11-21

**Authors:** Henock D. Yallew, Marek Vlk, Anurup Datta, Sebastian Alberti, Roman A. Zakoldaev, Jens Høvik, Astrid Aksnes, Jana Jágerská

**Affiliations:** † Department of Physics and Technology, UiT The Arctic University of Norway, NO-9037 Tromsø, Norway; ‡ Department of Electronic Systems, Norwegian University of Science and Technology (NTNU), NO-7491 Trondheim, Norway

**Keywords:** slot waveguide, MIR spectroscopy, laser absorption spectroscopy, methane, water adsorption, confinement factor, silicon-on-insulator

## Abstract

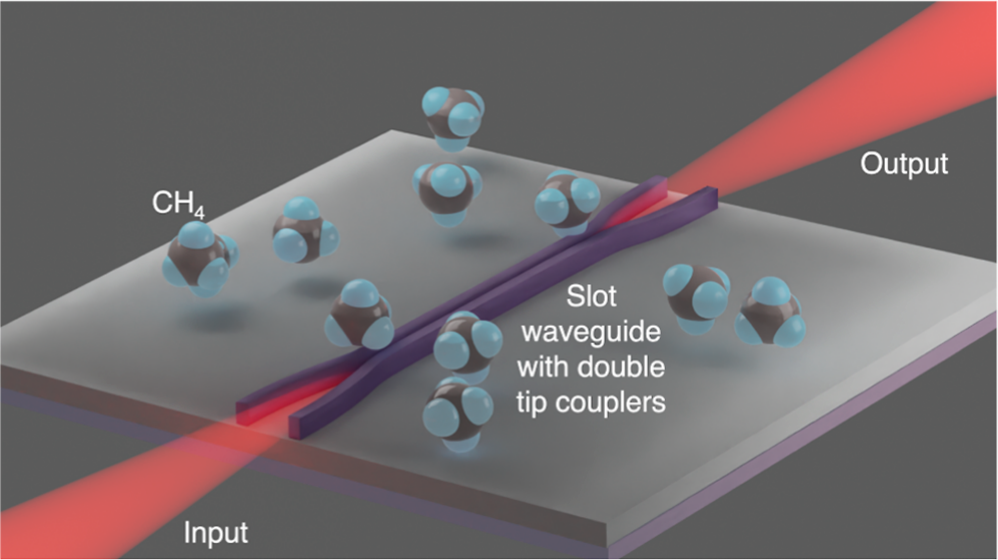

Hybrid integration of photonic chips with electronic and micromechanical circuits is projected to bring about miniature, but still highly accurate and reliable, laser spectroscopic sensors for both climate research and industrial applications. However, the sensitivity of chip-scale devices has been limited by immature and lossy photonic waveguides, weak light–analyte interaction, and etalon effects from chip facets and defects. Addressing these challenges, we present a nanophotonic waveguide for methane detection at 3270.4 nm delivering a limit of detection of 0.3 ppm, over 2 orders of magnitude lower than the state-of-the-art of on-chip spectroscopy. We achieved this result with a Si slot waveguide designed to maximize the light–analyte interaction, while special double-tip fork couplers at waveguide facets suppress spurious etalon fringes. We also study and discuss the effect of adsorbed humidity on the performance of mid-infrared waveguides around 3 μm, which has been repeatedly overlooked in previous reports.

## Introduction

Methane is a potent greenhouse gas with atmospheric concentrations in the parts per million range. As efforts to mitigate climate change increase, addressing methane emissions from various sources has become crucial in reducing their impact on global temperature rise. Its detection at sub-ppm levels, required both in climate research and for emission management by, e.g., the oil and gas industry, is currently performed by high-end spectroscopic techniques such as gas chromatography (GC), mass spectrometry (MS), or mid-infrared (MIR) laser absorption spectroscopy (LAS). Of all these, LAS has the advantage of being nondestructive, and it provides excellent sensitivity and specificity with relatively compact, portable platforms. Utilizing a LAS spectrometer based on a quantum cascade laser and a 76 m long multipass cell, methane has been traced down to impressive 12 ppt.^1^ Real-time monitoring of ^13^CH_4_, CH_3_D, or even double-substituted “clamped” methane isotopes in ambient air has been realized in combination with a preconcentration unit.^2,3^ NASA has deployed a LAS spectrometer with a sub-ppb limit of detection (LOD) on Mars to search for methane emissions from organic matter,^4^ and a shoebox-sized instrument with a LOD of 0.1 ppb has been recently demonstrated for drone-conducted measurements.^5^ However, when it comes to chip-scale devices, the state-of-the-art methane detection limits lie above 10 ppm, which translates into a 6 orders of magnitude difference in sensitivity compared to that of bulk instruments. Similar performance was observed for both LAS^6−8^ and conceptually different on-chip methane sensors such as Mach–Zehnder interferometers,^9^ pellistors, metal-oxide or electrochemical sensors,^10^ micro-GCs,^11^ and integrated Fourier transform IR spectrometers.^12^ Yet, small and cheap integrated methane sensors of sub-ppm resolution are much desired to upgrade current sensor networks and increase the data density to better quantify global emissions and constrain climate models.

Among LAS sensors, a leading example is IBM’s near-infrared (NIR) methane sensor based on a 20 cm long optical waveguide integrated on a silicon-on-insulator (SOI) photonic chip.^6,7^ However, the LOD of this sensor is still limited to only 100 ppm. Comparable or higher detection limits have been reported with MIR sensors including ridge SOI waveguide^8^ or chalcogenide waveguide sensors.^13,14^ In all cases, either weak light–analyte interaction, large propagation loss, or spurious fringe background in the transmission interfering with the spectral signal appeared to critically limit the detection sensitivity.

The sensitivity of optical sensors scales with the interaction length, which is constrained by the instrument size. While bulk instruments use multipass cells or cavities that can accommodate pathlengths of hundreds of meters, the best nanophotonic waveguide sensors have reported waveguide lengths of only several tens of centimeters. Even shorter pathlengths of 1–2 cm are seen in MIR waveguide sensors, where the achievable lengths are limited by immature materials and processing. Moreover, waveguides that have been used for sensing are almost exclusively based on planar, strip, or rib waveguides with poor field confinement in the gaseous analyte, typically 10 to 25%.^6,8,13,16,17^ Accordingly, the per-length sensitivity is reduced to a fraction of what is achieved along the same path length using a free-space beam that has the optical field entirely confined in air. Finally, integrated waveguides contain interfaces, such as end-facets and defects, which produce multiple reflections, creating etalon fringe patterns in the transmission. Such fringes interfere with the spectral signal, critically affecting the detection limits and long-term signal stability.^18,19^

In this work, we report on methane detection with a MIR slot waveguide, which simultaneously addresses the limitations of existing integrated solutions. Our design relies on a waveguide mode largely constrained to an air slot to maximize the light–matter interaction, while carefully designed double-tip couplers help to minimize etalon effects. Built into a LAS MIR spectroscopic setup, this sensor enables over 2 orders of magnitude improvement of LOD compared to the state-of-the-art waveguide sensors, detecting methane down to 300 ppb on a chip. At this sensitivity, it offers laboratory-quality measurements in a compact, field-capable format.

## Methods

**Figure 1:**
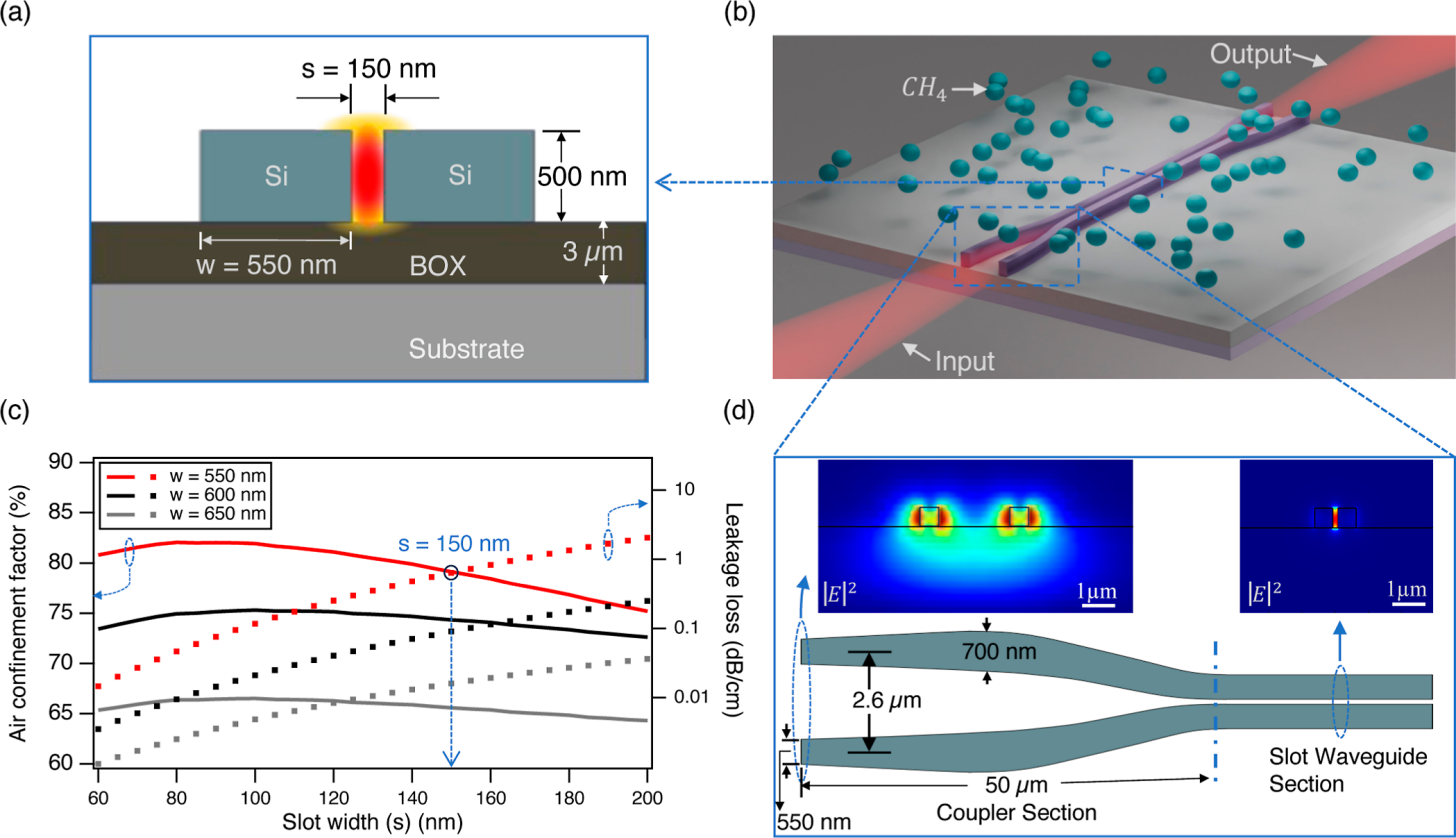
(a) Schematic view of the slot waveguide cross-section with superposed intensity distribution. (b) Layout of the sensor chip including double-tip couplers. The chip has a footprint of 1 cm^2^ and features 1.15 cm long straight waveguides terminated by 50 μm long couplers. (c) Simulated air confinement factor (solid lines) and leakage loss into the substrate (dotted lines) versus slot width *s* for different strip widths *w*. (d) Schematic of the double-tip coupler design with simulated mode profiles at the coupler facet and the slot waveguide cross-section.

A slot waveguide, first reported by Almeida et al.,^20^ is a unique waveguide geometry that supports hollow-core guiding. It consists of two higher-index waveguide core sections separated by a narrow slot filled with air or other low-index cladding material (Figure 1a). To satisfy boundary conditions, the horizontally oriented electric field of the fundamental TE mode undergoes discontinuities at the vertical sidewalls with stronger amplitude on the low refractive index side,^21^ leading to a field enhancement in the slot region.

Slot waveguides have gained popularity for applications where strong light–matter interaction between guided modes and low-index material is targeted, including switching, electrooptic modulation,^22^ and sensing.^23,24^

The light–matter interaction is, however, not scaled through the evanescent field overlap alone but through a quantity known as the confinement factor Γ. It can be shown that the external “air” confinement factor Γ_air_, i.e., outside the waveguide core, is equal to the product of the modal group index and the electric field energy density fraction residing in air^25−27^
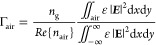
1where *n*_g_ is the modal group index, *n*_air_ the air refractive index, ϵ(*x*, *y*) the permittivity, and *E*(*x*, *y*) the electric field. Light absorption along a waveguide exposed to an absorbing environment can then be described by the modified Beer–Lambert law
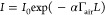
2with α being the bulk absorption coefficient of the analyte and *L* the physical length of the waveguide. To increase the absorption along a waveguide and thus the detection sensitivity, both the air confinement factor and the physical waveguide length, *L*, should be maximized. High Γ_air_ can be achieved by the use of dedicated waveguide geometry with optimized cross-sectional dimensions and high refractive index contrast between the core and the cladding.^26,27^ On the other hand, long physical pathlengths require low propagation loss. While scattering is the main source of loss in the NIR spectral region, a large part of the propagation loss in the MIR is attributed to absorption by the waveguide, either due to residual –OH or –NH bonds in oxides or nitrides, free-carrier absorption in semiconductors, residual impurities from fabrication (i.e., organic residues from photoresist or poor storage handling), or water present inside the material or adsorbed on the waveguide surface. Although material properties drive absorption loss in waveguides, we can design the mode to be primarily confined outside of the material and thus reduce the absorption effects. Finally, a large air confinement factor brings the effective index of the guided mode closer to the refractive index of the surrounding environment, leading to weaker reflections at waveguide defects and interfaces, which in turn reduces the etalon effects.

Slot waveguides with their large Γ_air_ simultaneously enable strong light–analyte interaction and mitigate both material absorption and fringing, making them well-adapted for sensing applications. Our design flow focused on maximizing Γ_air_ within the constraints given by available SOI wafer dimensions and the minimum pattern features achievable during processing.

Figure 1a,b illustrate the cross section of the slot waveguide and the top-view layout of the sensor. The sensor was designed using Lumerical to be compatible with commercial SOI wafers with a 500 nm thick silicon device layer on a 3 μm thick buried oxide (BOX) layer. The ambient air surrounding the waveguide serves as the top cladding and a region where gas molecules interact with the evanescent tail of the optical mode. The waveguide core width *w* and slot width *s* were optimized to support only the fundamental slot mode in TE polarization, prevent leakage loss through the BOX layer, and maximize Γ_air_. The Γ_air_ as a function of the slot width *s* is plotted for several core width values in Figure 1c, showing a broad and thus error-tolerant maximum for slot widths around 90 nm, which increases with decreasing *w*. The minimum feature size that could be successfully processed with our MIR-compatible 500 nm thick Si device layer was 140 nm. Adding a narrow margin, *s* was set to 150 nm and *w* to 550 nm to account for a steep rise in the leakage loss for *w* < 550 nm. This design provides Γ_air_ = 79% and leakage loss below 1 dB cm^–1^ (Figure 1c). More detail on waveguide simulation is provided in Supporting Information S1.

In order to facilitate light coupling in and out and reduce facet reflections, the slot waveguide was equipped on both ends with double-tip couplers inspired by the design of Nader et al.^28^ The double-tip couplers (Figure 1d) consist of two parallel arms with a cross section of 550 × 500 nm^2^ and a 2.6 μm separation at the chip facet. The superposed mode field of the coupler (Figure 1d) is largely delocalized and rather well matched to a Gaussian beam with a 7.5 μm diameter, generated by a 0.56 NA aspheric lens in our experimental setup. Away from the chip facet, both arms are first adiabatically widened to 700 nm to improve light confinement and reduce leakage loss into the substrate. The gap between the coupler arms is then gradually reduced and matched to the slot width, while the width of the arms is also adiabatically tapered into the design width *w* = 550 nm. As the mode propagates in the structure, the field profile is efficiently reshaped into a slot waveguide mode, as also shown in Figure 1d. With a total coupler length of 50 μm, we reduced the coupling loss from 8 to 3 dB and facet reflections from about 11 to 0.006% per interface, as compared to direct coupling to the slot waveguide.

### Waveguide Fabrication

**Figure 2:**
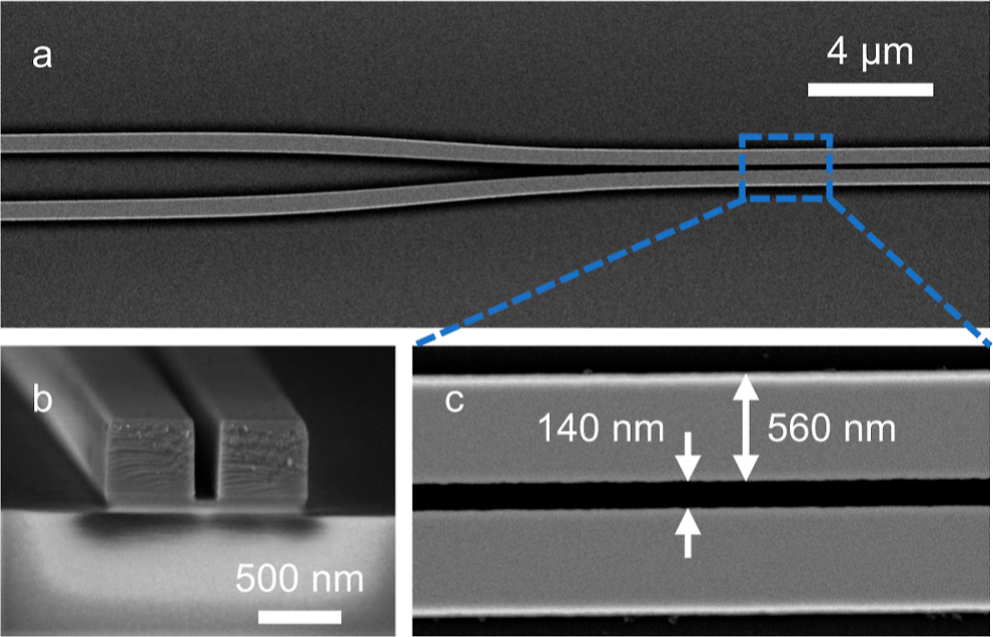
SEM images of the fabricated structure. (a) Tapering from the double-tip coupler to the slot waveguide. (b) Slot waveguide cross section and (c) top view.

The waveguide was fabricated via a standard CMOS process on a commercial SOI wafer (500 nm Si and 3 μm BOX) purchased from WaferPro. A 440 nm thick positive resist layer (CSAR62) was spin-coated onto a wafer sample and exposed with e-beam lithography (Elionix ELS-G100; 1 nA, 100 kV, and 300 μC cm^–2^). The waveguide pattern rendering was then finalized by developing it in AR 600–546 (amyl acetate). At this point, a mild O_2_ plasma descum step was applied to remove resisting residues from the developed areas. Next, the pattern was etched into the silicon layer with inductively coupled plasma–reactive ion etching (ICP–RIE; OIPT Plasmalab System 100 ICP–RIE 180) using CHF_3_/SF_6_ chemistry (50/7.5 sccm, 10 mTorr, 40 W RF, 600 W ICP, 20 °C). Any remaining resist was stripped off in the AR 600-71 (dioxolane) resist remover. The etching yielded well-defined couplers and Si slot waveguides with nearly vertical sidewalls of 88–89° captured in Figure 2. Structures with different lateral dimensions ranging from 550 to 650 nm for Si wire widths and from 140 to 180 nm for slot widths were fabricated.

Following the fabrication, the samples were annealed at 450 °C for 1 h to remove residual impurities such as organic leftovers from the fabrication as well as adsorbed water, resulting in reduced light absorption. Finally, the sample was cleaved in proximity to the couplers using a LatticeAx 225 cleaver with ±20 μm accuracy, and the optimal distance from the couplers was finely adjusted by polishing the facet to maximize the coupling efficiency.

## Results and Discussion

### Propagation Loss and Influence of Adsorbed Humidity

**Figure 3:**
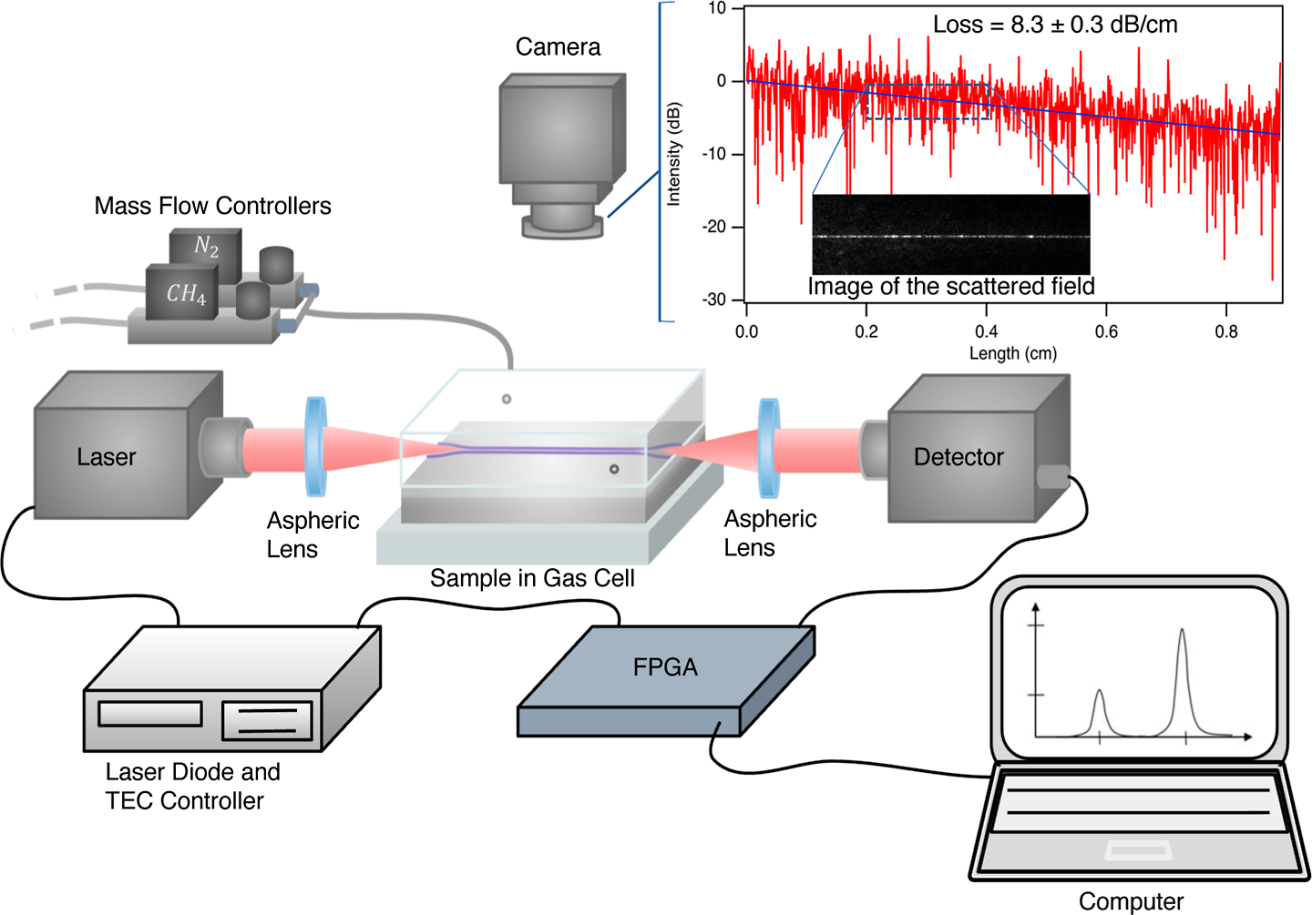
Schematic representation of the experimental setup. A collimated beam from the MIR ICL is focused onto the waveguide coupler with an aspheric lens. The MIR camera is used to aid the alignment and capture the scattered field from the waveguide for propagation loss evaluation. A representative scattered field image is shown as an inset, along with the intensity profile plot. The output beam is then collected with another aspheric lens and detected by the MCT detector. To perform CH_4_ spectroscopy and evaluate Γ_air_, the waveguide chip is enclosed in a gas cell equipped with transparent windows while mass flow controllers regulate the CH_4_ and N_2_ mixing ratio prior to injection. Finally, the PC and FPGA controlled laser tuning, detector signal acquisition, and HITRAN fitting.

The fabricated structures were first characterized in terms of propagation loss, coupling loss, and confinement factor using a combined imaging and spectroscopy setup shown in Figure 3. A MIR-distributed feedback interband cascade laser (DFB ICL, Nanoplus) electrically tunable across strong absorption lines of methane at 3270.4 nm was used as an light source. The laser beam was butt-coupled in and out of the waveguide chip by aspheric lenses with a numerical aperture of 0.56 and detected on an MCT photodetector (Vigo PVI-3TE-3.4). A MIR InSb camera (Telops) was used to aid the alignment and to monitor the light scattered out of plane from the waveguide to determine the propagation losses.

The signal decay along the waveguide was obtained from a series of top-view MIR images of the scattered light as shown in Figure 3. These images were corrected for the background signal, integrated over the waveguide cross-section, and plotted against the waveguide length. A linear fit of the data gave a propagation loss of 10.4 ± 0.3 dB cm^–1^, which exceeds expected cumulative losses from scattering, bottom cladding absorption, and substrate leakage that are anticipated within 1.4–5.9 dB cm^–1^ (see Supporting Information S1). We hypothesize that an important loss contribution comes from absorption by water adsorbed on the waveguide surface. A thin water layer is always created and maintained on hydrophilic surfaces such as silica or oxidized silicon surfaces due to atmospheric humidity.^29,30^ Since the methane absorption band at 3.25 μm overlaps with a strong water absorption band, the water can degrade the waveguide transmission. Assuming a liquid water extinction coefficient of *k* = 0.06^31^ and a 0.25 nm thick water monolayer, our simulation shows that the associated propagation loss can be as high as 14 dB cm^–1^ (Supporting Information S1). The impact of adsorbed water on the absorption loss can be controlled by flushing the surface of the waveguide with nitrogen/dry air or by keeping the sample heated up to reduce the surface coverage with water and thus absorption. After heating the sample at 115 ± 5 °C for 10 min and then keeping it in a nitrogen atmosphere, we successfully reduced the losses to 8.3 ± 0.3 dB cm^–1^ shown in Figure 3. Further loss decrease can be expected after prolonged degassing of both the sample and the entire gas flow system. A full experimental study investigating the dependence of the waveguide propagation loss on relative humidity is presented in Supporting Information S2.

### Confinement Factor

**Figure 4:**
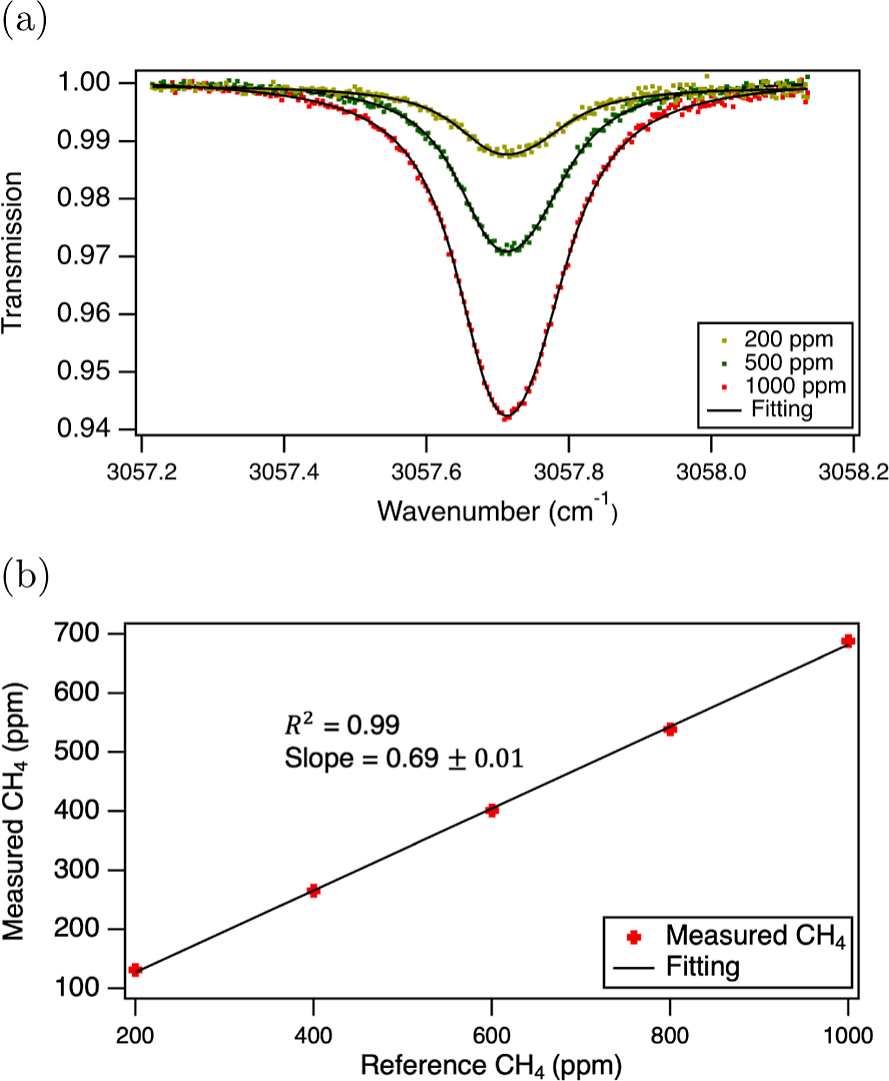
(a) Transmission spectra (dots) for 200, 500, and 1000 ppm reference concentrations and the corresponding HITRAN fits (black lines). (b) Measured concentrations plotted as a function of the reference concentration. Each point is an average of 300 steady-state spectra acquired at a rate of 1 Hz.

To experimentally determine the confinement factor, the methane absorption signal through a 1.15 cm long waveguide was measured and compared to free-space absorption for the same gas concentration. The utilized waveguide length was deemed ideal as it yielded a robust transmission signal within the dynamic range of our detector. It was also close to the theoretical optimal length of 0.8 cm expected to provide the highest detection sensitivity for methane in our experimental conditions (see Supporting Information S3 for details). During all further measurements, the chip was placed into a sealed gas cell (Figure 3) to create a controlled environment. Precise concentrations of methane between 0 and 1000 ppm in 200 ppm increments were prepared by diluting methane calibration gas (AGA, 999 ppm ± 2%) with pure nitrogen and introduced into the cell. To obtain the transmission spectra, the laser current was modulated with a linearly increasing ramp to electrically tune the emission wavelength by 2–3 nm around the center of the targeted methane absorption line, while the waveguide transmission was synchronously detected. The raw transmission spectra were first divided by the baseline, i.e., the transmission signal recorded with pure nitrogen, and finally fitted with the database spectra as shown in Figure 4a.

Figure 4b depicts the concentrations obtained from these fits with respect to the reference CH_4_ concentrations. The fitted concentrations show excellent linearity with respect to the reference, but they yield consistently lower values. This is expected as the lower concentrations reflect the scaling of the interaction in the waveguide relative to the free-space calibrated HITRAN (HIgh-resolution TRANsmission molecular absorption) database. The slope describing the ratio between the reference and measured values shown in Figure 4b determines the experimental waveguide confinement factor Γ_air_. The confinement factor value of 69% was found to be below the simulated 79%. The difference can be attributed to the uncertainty in structural dimensions and material parameters of the fabricated waveguides, unintentional collection of stray light from the chip substrate, influence of the coupler sections, and uncertainty in the reference gas concentration.

## Detection of Methane

**Figure 5:**
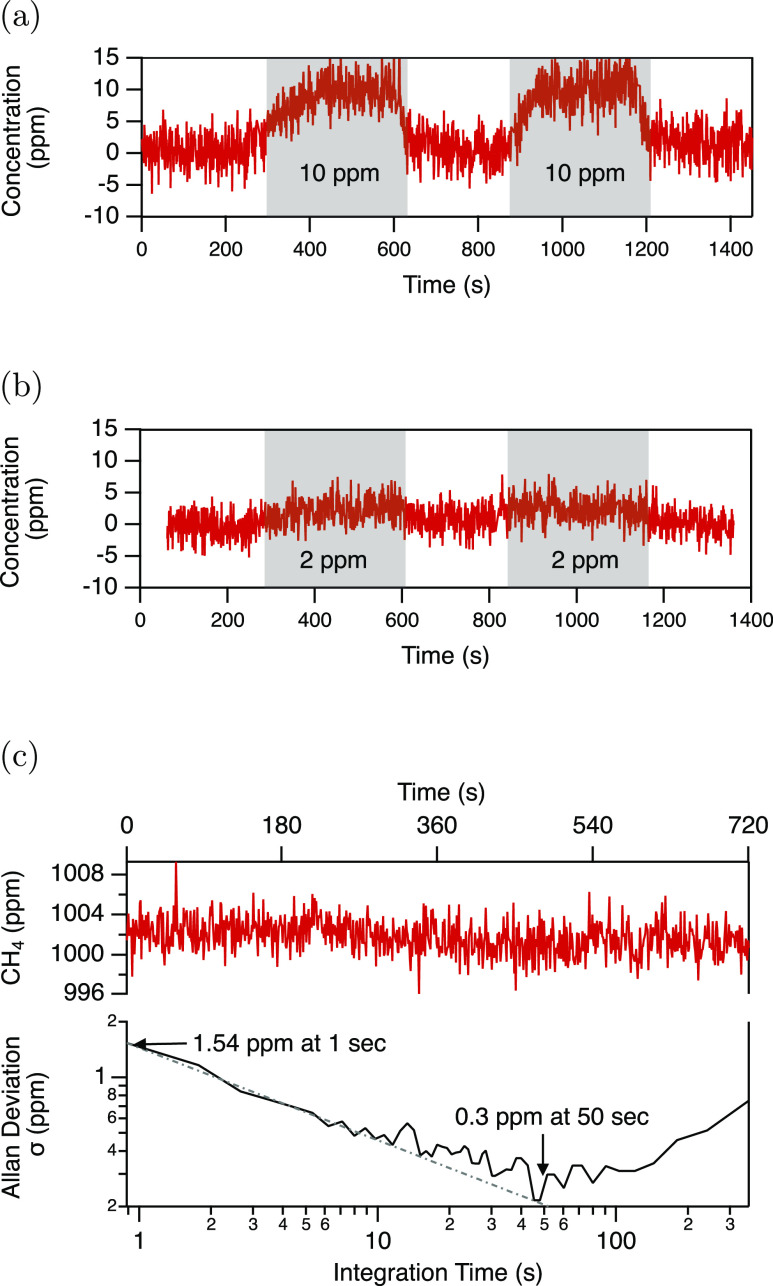
(a,b) 10 and 2 ppm of CH_4_ measured concentration, respectively. (c) 12 min time series with 1000 ppm of CH_4_ and the Allan deviation analysis for evaluation of the LOD and stability.

The confinement factor was then used to calibrate the sensor response to output actual methane concentration values. Figure 5a,b shows the sensor response to periodic methane concentration changes between 0 and 10 ppm and between 0 and 2 ppm, respectively. Each point in the time series corresponds to a concentration evaluated from 128 spectra recorded at 216 Hz, which were first averaged, baseline corrected, and fitted using calibrated HITRAN spectra. Spectral fitting with database spectra substantially reduces noise and thus LOD, improves measurement selectivity, and provides for “absolute calibration” of the measured data. In both cases, the sensor response clearly follows the concentration change trend, and it correctly quantifies the alternating concentrations. The sensor response to 2 ppm of CH_4_ indicates that this concentration approaches the detection limit of the sensor.

The LOD and stability of the system were rigorously evaluated through Allan deviation analysis from concentration data recorded for 1000 ppm of CH_4_ concentration during 12 min at room temperature. The measured concentration time series and the corresponding Allan–Werle plot are presented in Figure 5c. The standard deviation σ of 1.5 ppm was found for 1 Hz data, validating the sensor response in Figure 5b. At a 1 Hz detection rate, the detection sensitivity is mainly limited by detector noise and system vibrations that lead to variations in the coupling efficiency. After 50 s of averaging, a 1-σ LOD of 300 ppb is achieved. The sensor stability at this LOD is limited at present to about 150 s, beyond which the drifts in the setup gradually degrade the sensor performance.

Residual etalon fringes are the main source of long-term instability that eventually limits the performance as is common in most spectroscopic systems, including high-end, free-space-based instruments. Our double-tip couplers considerably reduce reflections to approximately 0.006% per facet. Accounting for the round-trip waveguide loss of 16 dB, the fringes should be effectively removed. Nevertheless, we still measured ripples in the transmission with amplitudes below 0.2%, which may be linked to reflections at defects and surface roughness. Since the LOD of 300 ppb corresponds to a transmission variation of 0.015%, even weak fringes play a critical role. Better LOD and long-term stability are expected after system integration, where the effects of mechanical misalignment and vibrations are limited and temperature control of the full system can be implemented. Digital etalon fringe removal algorithms demonstrated by the IBM group^18,32^ can be considered to push the LOD even further.

Finally, we should note that unlike free-space-based systems, our waveguide-based sensor is not entirely calibration-free due to the waveguide mode and, hence, confinement factor dependence on environmental parameters. In the previous analysis, we assumed that the waveguide confinement factor remained constant independently from gaseous sample composition and temperature. Such an assumption is valid only for stable environmental conditions with a simple gas matrix. Stabilization of the sensor limits the drift of etalons and will simultaneously prevent the variations of the confinement factor with temperature. However, recalibration of the sensor might still be necessary if high detection accuracy is needed in a large methane concentration range or at nonstandard operation conditions.

## Conclusions

In summary, leveraging nanophotonic SOI slot waveguides for methane detection allowed us to achieve 1-σ LODs of 1.54 ppm after only 1 s averaging and 300 ppb after 50 s at a 3270.4 nm wavelength. Such low LODs for methane are unprecedented with on-chip TDLAS, as Table 1 shows. The 300 ppb presents more than 2 orders of magnitude improvement from the state-of-the-art value of 78 ppm demonstrated by Zhao et al.^8^ or the 100 ppm reported by IBM in their seminal work.^6,7^ This suggests that the advantage of MIR operation combined with a dedicated waveguide design optimized toward strong light–matter interaction and reduction of etalons dominates over the waveguide path length, where IBM employed a 10× longer waveguide than us. For the reduction of etalons, our novel couplers practically eliminating facet reflections were essential alongside a low defect density in the fabricated waveguides.

**Table 1:** Comparison of Spectroscopic CH_4_ Waveguide Sensors in the Order of Increasing LOD^a^

ref	waveguide	1-σ LOD [ppm]	int. *t.* [s]	equivalent absorption	Γ [%]	*L* [cm]	λ [nm]	loss [dB cm^–^^1^]	method
this work	Si slot in SOI	1.54	1		69	1.15	3270.4	8.3 ± (0.3)	DAS
		0.3	50	1.70 × 10^–^^5^					
Zhao et al.^8^ 2022	Si rib in SOI	78	0.2	3.60 × 10^–^^4^	23.3	2	3291	0.71	WMS
Tombez et al^6^ 2017	Si strip in SOI	772	1		25.4	10	1650	2	DAS
		100	60	1.10 × 10^–^^4^					
Zhao et al.^8^ 2022	Si rib in SOI	155	0.2	7.20 × 10^–^^4^	23.3	2	3291	0.71	DAS
Bi et al^33^ 2023	Nb_2_O_5_ strip on SiO_2_	12 800	0.2		11.5^b^	2	3291	6.1	WMS
		349	61.2	7 × 10^–^^4^					
Su et al.^14^ 2019	chalcogenide strip on SiO_2_ (Ge_23_Sb_7_S_70_)	3100	13	2 × 10^–^^3^	12.5^b^	0.5	3310	8	NDIR
Han et al.^13^ 2016	chalcogenide strip on SiO_2_ (Ge_23_Sb_7_S_70_)	25000^c^	n/a	1.30 × 10^–^^2^	∼8^d^	2	3310	7	NDIR

a DAS—direct absorption spectroscopy, WMS—wavelength modulation spectroscopy, and NDIR—non-dispersive infrared (spectroscopy).

b These Γ values are theoretical.

c Authors have not specified the standard deviation magnitude, i.e., 1- or 3-σ.

d Authors have not reported whether this is the confinement factor or another measure such as power fraction.

To its advantage, the slot waveguide design allows for sharp bends and thus tight small-footprint patterning. This is facilitated by strong light confinement owing to the high refractive index contrast between Si and the surroundings. The active sample volume of the sensor is also remarkably low, as little as 1 picoliter, which could be exploited to open LAS to new applications in, for example, microbiology or organoid research. On the other hand, through the high refractive index contrast, the slot mode generates a strong field at the slot sidewalls, which leads to more pronounced effects of sidewall roughness and surface impurities than those in conventional strip or rib waveguides.

The propagation loss of our slot waveguide amounted to 8.3 ± 0.3 dB cm^–1^, which is largely attributed to water adsorbed on the waveguide surface. Water absorbs light significantly in the 2700–3500 nm range,^31^ and literature shows that waveguide performance in this band is underreported.^34,35^ Our work demonstrates how the effect of water is critical even without hitting the peak of its absorption band. We conditioned the waveguide chip by baking at 110–130 °C for 10 min and flushing with pure N_2_ prior to critical experiments to minimize the water influence. Nevertheless, our results suggest that a more rigorous approach is needed. In practical applications, the sample can be dried using a Nafion membrane dryer with desiccant, and waveguides can be functionalized or coated to prevent water adsorption. However, the coating homogeneity, roughness, thickness, and effects of the functional groups on the propagation loss would need to be rigorously characterized. Since most organic materials absorb light in the MIR, an ordinary hydrocarbon-based hydrophobic coating would likely aggravate the propagation loss.

The SOI platform employed in this work can be used up to approximately 4 μm, limited by the onset of strong SiO_2_ absorption. Within this spectral range, it can be applied to the trace-level detection of a variety of hydrocarbons, volatile organic compounds, NO_2_, H_2_S, H_2_O, SO_2_, and many other gases. The parasitic loss mechanisms of bottom cladding absorption and substrate leakage associated with the selected SOI wafer can be alleviated with other platforms. Alternatives like SOI wafers with thicker layers, Si on nonstandard substrates such as Al_2_O_3_^21,36^ or CaF_2_,^37^ or variations of Ge waveguides^38^ would then allow lower loss, longer interaction pathlengths, and, additionally, operation at longer MIR wavelengths.

Our slot-waveguide sensor tackled most challenges of waveguides for LAS, reaching a sub-ppm detection limit for methane for the first time. We can now detect minor increases from the atmospheric 1.9 ppm methane concentration,^39^ which is valuable for determining, e.g., landfill gas leaks. Yet, the sensor application goes beyond methane detection; arrays of slot waveguides optimized for a different analyte can realize a multigas sensor, measure gas fluxes, or provide for isotope-specific gas detection. Taking advantage of CMOS-compatible processing and abundant infrastructure, its practical deployment at low cost and large volumes, e.g., in smart sensor networks, is near.

## Supporting Information Available

Propagation loss—theory and simulation, experimental propagation loss, and theoretical LOD and the optimal waveguide length (PDF)
