# Supplementary material for: Sub-ppm Methane Detection with Mid-Infrared Slot Waveguides
Source: ACS Photonics. 2023 Nov 21;10(12):4282–9. doi: 10.1021/acsphotonics.3c01085 (PMC10740002; doi:10.1021/acsphotonics.3c01085)
Supplement: Supplementary file 1 — ph3c01085_si_001.pdf [file ph3c01085_si_001.pdf]

Sub-ppm Methane Detection with Mid-Infrared Slot Waveguides – Supporting Information

Henock D Yallew, Marek Vlk, Anurup Datta, Sebastian Alberti, Roman A Zakoldaev, Jens Høvik, Astrid Aksnes, and Jana Jágerská

Table of Contents

SUPPLEMENTARY 1: PROPAGATION LOSS – THEORY AND SIMULATION 2

SUPPLEMENTARY 2: EXPERIMENTAL PROPAGATION LOSS 5

SUPPLEMENTARY 3: THEORETICAL LIMIT OF DETECTION AND THE OPTIMAL WAVEGUIDE LENGTH 7

REFERENCES 9

Supporting Information 1: Propagation loss – theory and simulation

The primary causes of propagation loss in slot waveguides in the Mid-IR spectral range are attributed to 1) scattering, 2) mode leakage into the substrate, and 3) absorption within the waveguide material, dominated by absorption in the buried oxide (SiO_2) layer in SOI beyond wavelength of 3 μm. Furthermore, we argue that a 4th important loss mechanism around 3 μm is light absorption in a water layer adsorbed on waveguide's surface.

To theoretically investigate the leakage and absorption losses, a 2D cross-sectional model of the waveguide was developed using Ansys Lumerical simulation tool. A schematic of the waveguide in the model, with air as the top cladding, is shown in Fig. S1.

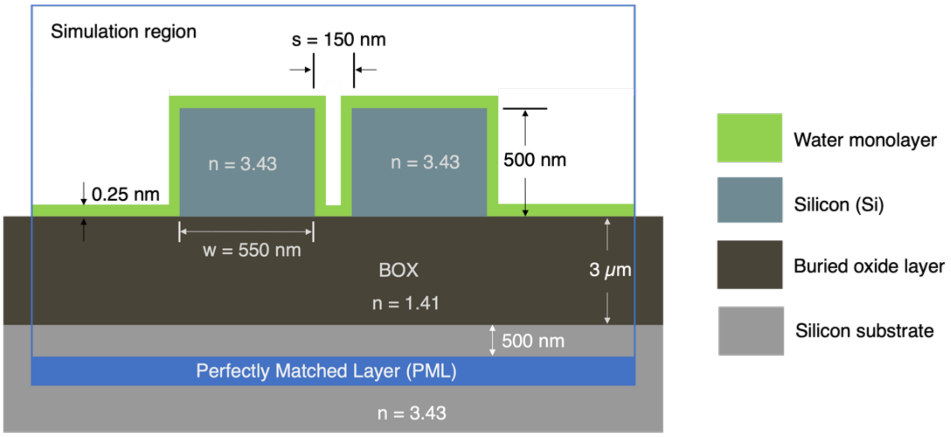

Fig. S1. A schematic depiction of the simulation model, with the simulation window extending over 12 μm laterally and 9.5 μm vertically. The PML boundary condition is positioned 500 nm below the buried oxide layer.

To evaluate the leakage loss, the simulation region employs a perfectly matched layer (PML) as the bottom boundary condition (BC), positioned within the silicon substrate to capture the portion of the optical mode that leaks into the substrate. The remaining boundaries are set to metal BC as no energy flows through them. The refractive indices of the materials are set to their respective real parts, and the region surrounding the slot is meshed with a 1 nm mesh size in both dimensions. Subsequently, the leakage loss was evaluated across a range of slot widths from 60 to 200 nm, and for silicon strip widths of 550, 600, and 650 nm. Simulation results in Fig. S2a show that leakage becomes substantial (larger than 1 dB cm^{-1}) for $w \leq 550$ nm and $s \geq 160$ nm.

To calculate the loss through absorption in the bottom cladding, the SiO_2 absorption was modelled by setting the refractive index imaginary part, i.e., extinction coefficient, to 3.9×10^{-6}, which corresponds to absorption coefficient of 0.15 cm^{-1} at 3270.4 nm wavelength [1]. The contribution of SiO_2 absorption (Fig. S2b) was subsequently determined by subtracting the leakage loss from the total loss obtained using the SiO_2 extinction coefficient.

Balancing losses and high confinement factor (Fig. 1c in the manuscript), the cross-sectional dimensions were set to s = 150 nm for the slot and w = 550 nm for the strip width. These dimensions lead to the leakage loss of 0.7 dB cm^{-1} and SiO_2 absorption loss of 0.2 dB cm^{-1} according to Fig. S1. In total, 0.9 dB cm^{-1} loss can be expected theoretically if scattering and water absorption are disregarded.

a)

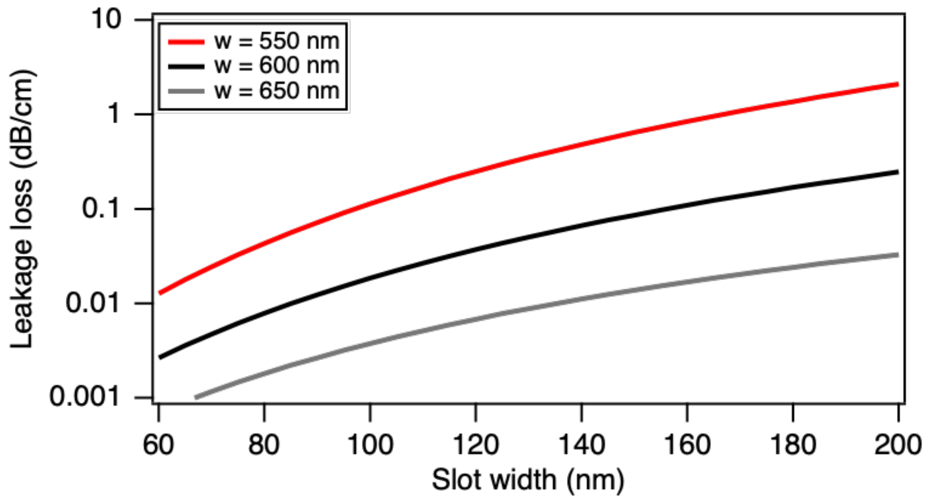

b)

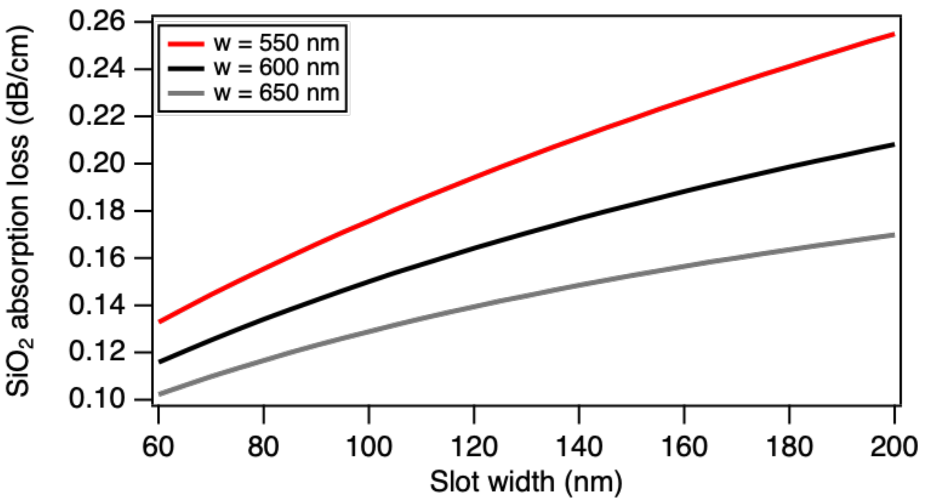

Fig. S2. a) the simulated leakage loss and b) the simulated SiO_2 absorption loss. Both losses were evaluated for strip widths, w = 550, 600, and 650 nm and exhibit an increase as the slot width widens and a decrease when the strip width is increased.

Scattering represents an important loss contribution. However, simulating scattering loss requires a detailed knowledge of surface roughness and imperfections, which experimental characterization is complex and beyond the scope of this paper. Instead, we attempt to constrain the scattering loss based on experimental measurements provided in literature in the near-IR spectral region: SOI waveguides with air in the slot report at 1550 nm losses 10–100 dB cm^{-1}, where the loss is predominantly due to

scattering [2]–[4]. The scattering loss follows Rayleigh's $1/\lambda^4$ scaling law, therefore, at 3270.4 nm we can expect scattering loss 0.5–5 dB cm^{-1}. The total waveguide propagation loss excluding water absorption can thus be expected between 1.4 and 5.9 dB cm^{-1}. The lowest value exactly corresponds to the experimental value reported in [5], where loss of 1.4 dB cm^{-1} was measured in slot waveguides of comparable dimensions to ours at the wavelength of 3.8 μm (this wavelength lies outside of the water absorption band).

Finally, the impact of the adsorbed water was simulated by introducing a 0.25 nm thin layer of water on the slot waveguide's surface with an extinction coefficient of 0.06 [6]. All other materials were assumed lossless. This simulation was carried out using the optimized dimensions, where the slot and strip widths are 150 and 550 nm, respectively. The water layer was meshed with 0.001 nm mesh size, providing sufficient resolution for even such thin, subnanometer layer. To isolate the effect of water absorption, the leakage loss was again subtracted from the total loss.

A monolayer is taken as a reference surface coverage state for a hydrophilic surface at moderate (25%) to low humidity (<10%). This is supported by several experimental works done with different techniques including mechanical resonators, FTIR, and adsorption measurements on powder. It is worth noting that a lower coverage than a monolayer is expected at low humidity, while more than one monolayer can form at humidity above 50%. The choice of 0.25 nm thickness for a monolayer was taken as an estimate from reported values ranging from 0.23 to 0.28 nm and in close relation to the tabulated value of the water molecule diameter [7], [8]. Nevertheless, the selected values of thickness and extinction coefficient should be taken as rough estimates. Other factors need to be considered: Surface roughness increases the surface area and the amount of water; The degree of hydroxylation of silica and the thickness of the naturally oxidized layer on silicon alter the hydrophilicity of the surface and the extinction coefficient of the material; Finally, water molecules on the surface exist in an intermediate state between liquid and ice. The first layer is close to ice while the subsequent layers are closer to liquid. Changes in the extinction coefficient and surface density of water molecules on the surface can be therefore expected [9]–[11].

The propagation loss associated with the water monolayer amounts to 14 dB cm^{-1} according to the simulation. However, this number may not accurately reflect the loss as discussed above: It is a function of ambient humidity, surface roughness, and the sample history (processing and storage). Nevertheless, the simulation shows that adsorbed water must be considered in the 3 μm range as it represents an important loss mechanism, seriously affecting and potentially limiting light propagation in photonic integrated circuits.

In conclusion, the cumulative value of all simulated losses gives 15.4–19.9 dB cm^{-1} when a 0.25 nm water monolayer is considered on the slot waveguide surface.

Supporting Information 2: Experimental propagation loss

In this section, we experimentally investigate propagation losses in the fabricated slot waveguide (refer to Fig. 2) as a function of atmospheric humidity. We also study the reduction of the propagation loss by sample heating, which is expected to accelerate water removal from the sample surface.

The applied methodology of propagation loss measurement is detailed in the manuscript (section 4.1 and Fig. 3). During the measurement, the waveguide chip was placed in a sealed gas cell as described in section 4.2. Three different conditions of relative humidity (RH) were generated in the cell: 70–75% RH by flowing nitrogen through a gas bubbler submerged in water; 21% RH of ambient air; and 2.5% RH with dry nitrogen. Humidity in the cell was measured using an independent humidity sensor (Sensirion, SHT41-AD1F). Residual humidity of 2.5% registered in the presence of dry nitrogen is associated with system degassing. In the final step, the waveguide chip was heated to 115 ± 5 °C for 10 min on a hot plate and then placed back to the nitrogen atmosphere in the gas cell.

The summary of our experimental findings is presented in Fig. S3. The waveguide kept at 70–75% RH for 1 min showed losses of 13.8 ± 0.4 dB cm^{-1}. Exposure to ambient conditions at 21% RH decreased the loss to 10.4 ± 0.3 dB cm^{-1}, while introducing dry nitrogen into the gas cell led to a further loss decrease to 9.7 ± 0.3 dB cm^{-1}. Finally, the heat treatment reduced the losses down to 8.3 dB cm^{-1}.

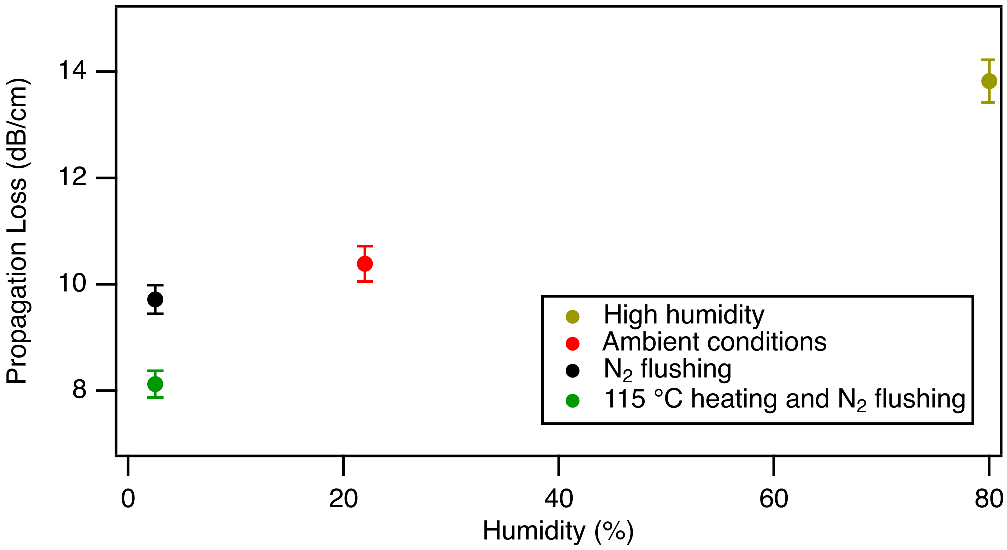

Fig. S3. Waveguide propagation losses at different humidity.

A clear dependence of the losses on RH is observed as shown in Fig. S3. The total loss decreases by 5.5 dB from high humidity to dry conditions, while further loss decrease can be expected after prolonged degassing of both the sample and the gas flow system. This experimental data supports our discussion in Supplementary 1, demonstrating that a minimal presence of water (a fraction of a monolayer) is detrimental for the total loss of our waveguides at the studied wavelength. At the same time, the experimental result indicates that the assumption of 1 full water monolayer considered in the

theoretical model in Supplementary 1 qualitatively explains but slightly overestimates the effect of absorbed water, which can be linked both to the uncertainty in in the assumed water absorption coefficient and the nature of water adsorption on our particular surface (silica hydroxylation on the surface and kinetic parameters of water adsorption).

Supporting Information 3: Theoretical limit of detection and the optimal waveguide length

The limit of detection (LOD) of a spectroscopic waveguide sensor depends primarily on the waveguide properties (length L, confinement factor Γ, propagation loss α_{prop}, coupling efficiency E), the laser properties (power P_0), and the detector properties (noise equivalent power – NEP). Analytical expression for LOD dependence on the above quantities can be derived from Lambert-Beer law [12]:

$$C_{\min} = \frac{-\ln\left[1 - \frac{\mathrm{NEP}}{P_0 E^2 \exp\left(-\alpha_{\mathrm{prop}} L\right)}\right]}{\Gamma \varepsilon L}$$

Here, ε represents a weighted methane absorption coefficient in units of $\mathrm{ppm}^{-1}\mathrm{cm}^{-1}$. In our setup, we employed a laser with output power up to 1 mW (measured after collimating and focusing optics), a detector with NEP of 1.6×10^{-7} W (Vigo PVI-3TE-3.4 with PIP-DC-20M preamplifier, NEP measured experimentally), coupling loss is assumed 3 dB ($E = 0.5$), $\alpha_{prop} = 8.3$ dB $\mathrm{cm}^{-1} = 1.9\ \mathrm{cm}^{-1}$, $\Gamma = 0.69$, and $\varepsilon = 5 \times 10^{-5}\ \mathrm{ppm}^{-1}\ \mathrm{cm}^{-1}$ was obtained from HITRAN database for CH_4 peak at 3057.7 cm^{-1}/ 3270.4 nm.

The dependence of LOD on L shows a broad minimum at $L = 0.5$ cm, which can be considered the "optimal waveguide length" L_{opt}, leading to a minimal LOD of 96 ppm as shown in Fig. S4 below.

In real systems, however, the dynamic range of the detector must be considered. The detector used in our study saturates at 0.06 mW, meaning the laser power must be reduced below 0.06 mW before reaching the detector. For $L_{opt} = 0.5$ cm, the power out-coupled from the waveguide calculated as $P_0\, E^2 \exp(-\alpha_{prop}\, L_{opt})$ is 0.1 mW, saturating the detector. Power reduction can be done either by decreasing P_0 (e.g., using an intensity filter), or by extending L beyond L_{opt} and reducing the power through propagation loss. Both methods will lead to an increase in the LOD, however, it is evident from Fig. S4 that extension of the waveguide length affects the LOD less significantly than reduction of the power P_0. At $L = 0.8$ cm, the power at the detector is low enough to not saturate the detector, and the LOD increases by only 10 ppm. Waveguide length of 0.8 cm can thus be effectively considered the true optimal length.

Our waveguide length of 1.15 cm is longer but still close to the true optimal length; it slightly increases the optimal LOD but it allows for a sufficient margin, reducing the risk of saturating the detector.

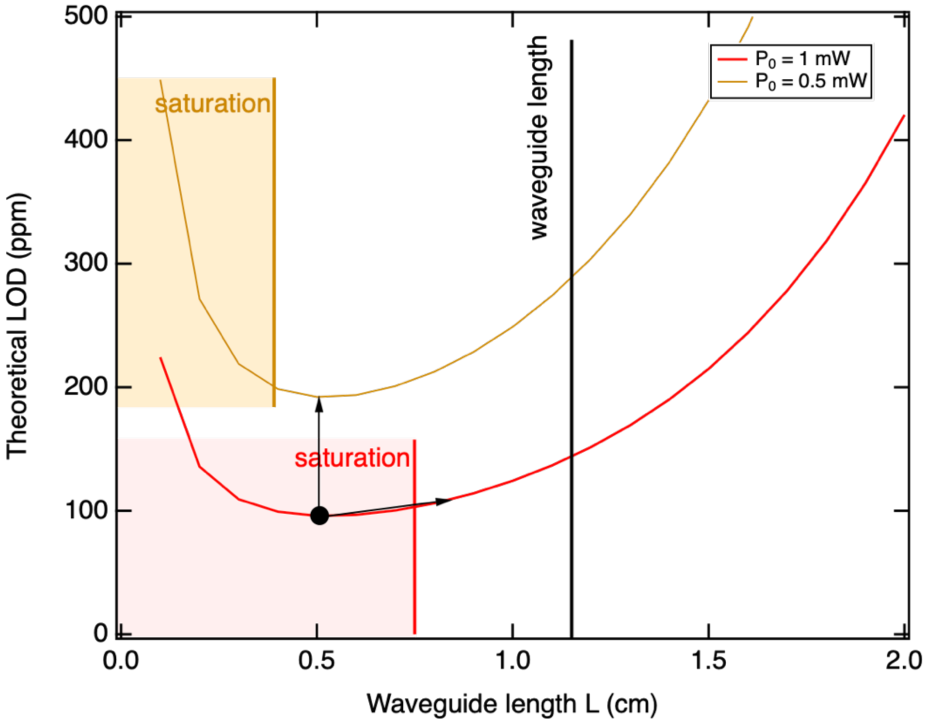

Fig. S4: Visualization of the LOD as a function of L, including the margins set by the detector saturation power.

It must be noted that although such theoretical optimal length is a rather precise model, the theoretical LOD is only indicative and cannot be directly compared to our experimental result due to the following:

- The calculated LOD represents the LOD at the acquisition rate r (in our case 216 Hz); averaging reduces the LOD, e.g., 1-second LOD is reduced by $\sqrt{r}$.
- Further LOD reduction is achieved through dedicated signal processing such as least-square fitting of the full absorption profile adopted in our work. The above theory assumes recording peak amplitude at one point in the spectrum.
- In real systems, the recorded noise is typically larger than the detector NEP, dominated by the laser noise, spectral fringes, noise due to mechanical vibration etc. Such noise sources are difficult to predict and model.

References

[1] R. Kitamura, L. Pilon, and M. Jonasz, "Optical constants of silica glass from extreme ultraviolet to far infrared at near room temperature," *Appl. Opt.*, vol. 46, no. 33, p. 8118, Nov. 2007, doi: 10.1364/AO.46.008118.

[2] T. Alasaarela *et al.*, "Reduced propagation loss in silicon strip and slot waveguides coated by atomic layer deposition," *Opt. Express*, vol. 19, no. 12, p. 11529, Jun. 2011, doi: 10.1364/OE.19.011529.

[3] M. Scullion, T. Krauss, and A. Di Falco, "Slotted Photonic Crystal Sensors," *Sensors*, vol. 13, no. 3, pp. 3675–3710, Mar. 2013, doi: 10.3390/s130303675.

[4] Y. Wang, M. Kong, Y. Xu, and Z. Zhou, "Analysis of scattering loss due to sidewall roughness in slot waveguides by variation of mode effective index," *J. Opt.*, vol. 20, no. 2, p. 025801, Feb. 2018, doi: 10.1088/2040-8986/aa9f8f.

[5] J. Soler Penades, A. Khokhar, M. Nedeljkovic, and G. Mashanovich, "Low Loss Mid-Infrared SOI Slot Waveguides," *IEEE Photonics Technol. Lett.*, pp. 1–1, 2015, doi: 10.1109/LPT.2015.2414791.

[6] G. M. Hale and M. R. Querry, "Optical Constants of Water in the 200-nm to 200-\si{\micro\metre} Wavelength Region," *Appl. Opt.*, vol. 12, no. 3, p. 555, Mar. 1973, doi: 10.1364/AO.12.000555.

[7] P. Schatzberg, "Molecular diameter of water from solubility and diffusion measurements," *J. Phys. Chem.*, vol. 71, no. 13, pp. 4569–4570, Dec. 1967, doi: 10.1021/j100872a075.

[8] P. B. Miranda, L. Xu, Y. R. Shen, and M. Salmeron, "Icelike Water Monolayer Adsorbed on Mica at Room Temperature," *Phys. Rev. Lett.*, vol. 81, no. 26, pp. 5876–5879, Dec. 1998, doi: 10.1103/PhysRevLett.81.5876.

[9] M. L. Hair and W. Hertl, "Adsorption on hydroxylated silica surfaces," *J. Phys. Chem.*, vol. 73, no. 12, pp. 4269–4276, Dec. 1969, doi: 10.1021/j100846a039.

[10] D. B. Asay and S. H. Kim, "Evolution of the Adsorbed Water Layer Structure on Silicon Oxide at Room Temperature," *J. Phys. Chem. B*, vol. 109, no. 35, pp. 16760–16763, Sep. 2005, doi: 10.1021/jp053042o.

[11] P.-O. Theillet and O. N. Pierron, "Quantifying adsorbed water monolayers on silicon MEMS resonators exposed to humid environments," *Sens. Actuators Phys.*, vol. 171, no. 2, pp. 375–380, Nov. 2011, doi: 10.1016/j.sna.2011.09.002.

[12] J. Shim, J. Lim, D.-M. Geum, B. H. Kim, S.-Y. Ahn, and S. Kim, "Tailoring bolometric properties of a $TiO_x/Ti/TiO_x$ tri-layer film for integrated optical gas sensors," *Opt. Express*, vol. 29, no. 12, p. 18037, Jun. 2021, doi: 10.1364/OE.427147.
